# Targeting PRMT5 through PROTAC for the treatment of triple-negative breast cancer

**DOI:** 10.1186/s13046-024-03237-y

**Published:** 2024-11-30

**Authors:** Yaxun Guo, Yuzhan Li, Zhongmei Zhou, Lei Hou, Wenjing Liu, Wenlong Ren, Dazhao Mi, Jian Sun, Xueqin Dai, Yingying Wu, Zhuo Cheng, Tingyue Wu, Qianmei Luo, Cong Tian, Fubing Li, Zhigang Yu, Yihua Chen, Ceshi Chen

**Affiliations:** 1 Department of Breast Surgery, The Second Hospital of Shandong University, Jinan, 250033 China; 2 Shanghai Frontiers Science Center of Genome Editing and Cell Therapy, Shanghai Key Laboratory of Regulatory Biology, Institute of Biomedical Sciences, School of Life Sciences, East China Normal University, Shanghai, 200241 China; 3 The School of Continuing Education, Kunming Medical University, Kunming, 650500 China; 4 Department of Breast Disease, Henan Breast Cancer Center, Affiliated Cancer Hospital of Zhengzhou University & Henan Cancer Hospital, Zhengzhou, 450008 China; 5 Yunnan Key Laboratory of Breast Cancer Precision Medicine, Yunnan Cancer Hospital, The Third Affiliated Hospital of Kunming Medical University, Peking University Cancer Hospital Yunnan, Kunming, 650118 China; 6 School of Life Science, University of Science & Technology of China, Hefei, 230027 China; 7 Yunnan Key Laboratory of Animal Models and Human Disease Mechanisms, Kunming Institute of Zoology, Chinese Academy of Sciences, Kunming, 650201 China; 8 Yunnan Key Laboratory of Breast Cancer Precision Medicine, Academy of Biomedical Engineering, Kunming Medical University, Kunming, 650500 China; 9 Department of Pathology, The First Affiliated Hospital of Kunming Medical University, Kunming, Yunnan, 650032 China; 10 Institute of Translational Medicine of Breast Disease Prevention and Treatment, Shandong University, Jinan, 250033 China; 11 Shandong Provincial Engineering Laboratory of Translational Research on Prevention and Treatment of Breast Disease, Jinan, 250033 China; 12 School of Pharmaceutical Sciences, Yunnan Key Laboratory of Pharmacology for Natural Products, Kunming Medical University, Kunming, 650500 China; 13 Yunnan College of Modern Biomedical Industry, Kunming Medical University, Kunming, 650500 China

**Keywords:** PROTAC, PRMT5, KLF5, TNBC

## Abstract

**Background:**

Triple-negative breast cancer (TNBC) is currently the most aggressive subtype of breast cancer, characterized by high heterogeneity and strong invasiveness, and currently lacks effective therapies. PRMT5, a type II protein arginine methyltransferase, is upregulated in numerous cancers, including TNBC, and plays a critical role, marked it as an attractive therapeutic target. PROTAC (Proteolysis Targeting Chimeras) is an innovative drug development technology that utilizes the ubiquitin-proteasome system (UPS) to degrade target proteins, which is characterized by higher activity, enhanced safety, lower resistance, and reduced toxicity, offering significant value for clinical translation.

**Methods:**

This study utilizes the PROTAC technology to develop potential degraders targeting PRMT5 in vitro and in vivo.

**Results:**

Through the design, synthesis and screening of a series of targeted compounds, we identified YZ-836P as an effective compound that exerted cytotoxic effects and reduced the protein levels of PRMT5 and its key downstream target protein KLF5 in TNBC after 48 h. Its efficacy was significantly superior to the PRMT5 PROTAC degraders that had been reported. YZ-836P induced G1 phase cell cycle arrest and significantly induced apoptosis in TNBC cells. Additionally, we demonstrated that YZ-836P promoted the ubiquitination and degradation of PRMT5 in a cereblon (CRBN)-dependent manner. Notably, YZ-836P exhibited pronounced efficacy in inhibiting the growth of TNBC patient-derived organoids and xenografts in nude mice.

**Conclusions:**

These findings position YZ-836P as a promising candidate for advancing treatment modalities for TNBC.

**Trial registration:**

Ethics Committee of Yunnan Cancer Hospital, KYCS2023-078. Registered 7 June 2023.

**Supplementary Information:**

The online version contains supplementary material available at 10.1186/s13046-024-03237-y.

## Background

Breast cancer is the most prevalent tumor among women worldwide, with an increasingly younger age of onset, posing a severe threat to their physical and mental health. Triple-negative breast cancer (TNBC) is characterized by the absence of estrogen receptor (ERα), progesterone receptor (PR), and human epidermal growth factor receptor 2 (HER2) expression. This leads to rapid disease progression, high recurrence rates, a tendency for distant metastasis, and a lack of effective targeted therapies, making it one of the subtypes with a poor prognosis [1]. Patients with stage I-III TNBC have higher rates of recurrence and mortality [2], while stage IV patients have shorter overall survival [3, 4]. Currently, chemotherapy remains the main treatment for TNBC [5]; however, it has drawbacks such as drug resistance, severe side effects, and varying treatment responses [6]. Therefore, there is a critical need to pursue novel therapeutic targets and targeted agents for TNBC.

Arginine-methylated proteins play a crucial role in various cellular processes necessary for maintaining tissue homeostasis and disease phenotypes. Protein arginine methyltransferase 5 (PRMT5), categorized as a type II protein arginine methyltransferase enzyme, is known for its ability to symmetrically dimethylate several substrates, including histones (such as H4R3, H3R2, H3R8, and H2AR3) as well as non-histone proteins, like c-Myc, CyclinD1, Slug, KLF4, KLF5, etc [7–11]. The process of arginine methylation governed by PRMT5 plays a significant role in regulating critical biological pathways, such as cell growth, apoptosis, stemness, and motility [12]. Notably, in various tumors including lung, breast, stomach, and liver etc., PRMT5 is overexpressed, which underscores its implication in cancer pathology [13]. In breast cancer, particularly, the expression level of PRMT5 is markedly elevated when compared to that in normal breast tissue [14]. Targeted inhibition of PRMT5 has demonstrated effective suppression of the growth, proliferation, migration, and invasion of breast cancer cells [15]. Moreover, PRMT5’s ability to methylate KEAP1 and inhibit ferroptosis presents a challenge to the efficacy of immunotherapy in TNBC [16]. Our research has unveiled a novel mechanism by which PRMT5 facilitates the progression of TNBC by methylating KLF5; thereby reducing its phosphorylation and ubiquitin-mediated degradation [11]. Currently, there are no approved drugs targeting PRMT5. Most PRMT5 inhibitors are still in the early stages of clinical trials with limited clinical application due to numerous adverse events [17]. However, PROTAC (Proteolysis Targeting Chimeras) may provide a new strategy for developing PRMT5 degraders.

PROTAC is an emerging drug development technology that utilizes the ubiquitin-proteasome system (UPS) to degrade target proteins. PROTACs consist of heterobifunctional molecules composed of two ligands: one recruits and binds to an E3 ubiquitin ligase while the other binds to the target protein of interest (POI). These two ligands are connected by a linker forming a “trimeric” structure-target protein ligand-linker-E3 ligand [18]. This ternary complex brings the target protein and E3 ligase into close proximity, inducing the ubiquitination of the target protein, which is then recognized and degraded by the proteasome [19, 20].

Compared to traditional small-molecule drugs, PROTACs possess unique capability in targeting and degrading proteins devoid of active sites, such as scaffold proteins. This attribute markedly broadens the spectrum of potential therapeutic targets. Additionally, PROTAC molecules are not required to maintain prolonged binding with the target protein to initiate its degradation and achieve its complete functional abrogation. This characteristic could potentially circumvent the issue of drug resistance frequently encountered with small-molecule inhibitors [21]. PROTAC technology also offers several advantages, including a broader scope of action, higher activity, better selectivity, enhanced safety, lower resistance, and reduced toxicity [22]. Currently, PROTAC technology has shown tremendous potential and promise in various fields, including cancer [23–25], immune disorders [26, 27], and neurodegenerative diseases [28]. In the case of breast cancer, a series of PROTAC compounds have been identified, such as ARV-471, which targets ER degradation for the treatment of locally advanced or metastatic ER-positive, HER2-negative breast cancer patients [29, 30]. In the VERITAC trial, ARV-471, as a monotherapy for advanced or metastatic ER+/HER2- breast cancer patients, demonstrated good tolerability and a 38% clinical benefit rate (including complete response, partial response, or disease stabilization for more than 24 weeks). These findings accentuate the efficacy and therapeutic potential of PROTAC technology in breast cancer treatment, heralding a new era of targeted molecular therapies [31].

In 2020, Shen et al. first reported the PROTAC compound MS4322, which targeted PRMT5. The molecular structure of this compound combined the PRMT5 inhibitor EPZ015666 with the von Hippel-Lindau (VHL) E3 ligase ligand, (*S*,*R*,*S*)-AHPC-Me (VHL-2). MS4322 effectively reduced PRMT5 protein levels in the estrogen receptor (ER) + breast cancer cell line MCF-7 in concentration-, time-, PRMT5-, VHL-, and proteasome-dependent manners [32]. Given the high toxicity of PRMT5 inhibitors currently under clinical investigation, the discovery of MS4322 may provide new insights into developing safer and more effective PRMT5 inhibitors. However, the degradation effect of MS4322 was modest, and it took 8 days to achieve the optimal degradation effect of PRMT5. Therefore, it is necessary to develop more efficient PRMT5 PROTACs for the treatment of TNBC.

In this study, we identified YZ-836P as a CRBN-recruiting PRMT5 degrader which exerts cytotoxic effects in TNBC. YZ-836P promotes the ubiquitination of PRMT5 by recruiting cereblon (CRBN), culminating in the proteasomal degradation of PRMT5. This mechanism provides a crucial theoretical foundation and experimental evidence for the advancement of PROTAC-based therapeutics targeting PRMT5 and analogous proteins. Additionally, we demonstrated the significant tumor growth inhibitory effects of YZ-836P using patient-derived organoids (PDOs) and xenograft models of TNBC. These findings underscore the potential of YZ-836P as a promising candidate for the treatment of TNBC.

## Methods

### Compound synthesis

The detailed synthesis process and characterization of the compound YZ-836P was described in Figure S1.

### Cell culture and treatment

All cell lines used in this study were purchased from American Type Culture Collection (ATCC, Manassas, VA, USA) and validated via short tandem repeat (STR) analysis. Different cells were cultured in different media, as detailed in Table S1. All cells were maintained at 37 °C in an incubator with 5% CO_2_.

### Cell viability assays

A specific amount of cells were seeded into a 96-well plate. After adhesion, the cells were treated with a compound for a predetermined time. Then, 100 µL of 10% trichloroacetic acid (TCA) were added to each well and left it overnight at 4 °C to fix the cells. Afterwards, we washed off the TCA with deionized water (dH_2_O) and allowed the wells to dry. Next, the cells were stained the fixed with Sulforhodamine B (SRB) solution for 30 min, washed with 1% acetic acid and dried. Finally, 100 µL of Tris base was added to dissolve the SRB dye and the absorbance at 530 nm was measured using a microplate reader (Infinite M200 Pro, Tecan).

### Western blotting (WB)

First, cells were washed with PBS and added an appropriate amount of RIPA lysis buffer containing protease and phosphatase inhibitors to extract proteins on ice for 30 min. The cell lysates were collected and centrifuged. The protein was quantified using the Pierce™ BCA Protein Assay Kit (23225, Thermo scientific), then mixed the samples with 4×sodium dodecyl sulfate (SDS) loading buffer proportionally and boiled at 98 °C for 10 min. The samples were separated via SDS-PAGE and transferred onto a PVDF membrane. The membrane was blocked with 5% non-fat milk for 1 h and incubated overnight with the primary antibody at 4 °C, then, incubated with horseradish peroxidase-conjugated secondary antibody at room temperature for 1 h. Signals was detected using an enhanced chemiluminescence reagent (UE, S6009) through ImageQuant LAS4000 (GE, Germany). **Table S2** listed the antibodies used in this study.

### Clonogenic assays

3,000 cells were seeded into a 6-well plate and incubated with varying concentrations of a compound. After 2 days, the medium was replaced with fresh culture medium and the cells were continued to culture for approximately 2 weeks. The cells were fixed using 4% paraformaldehyde, then stained with 0.1% crystal violet solution. Finally, individual colonies containing more than 50 cells were counted under a microscope.

### EdU assays

We assessed cell proliferation using the 5-ethynyl-20-deoxyuridine (EdU) assay kit (HC1010, US Everbright Inc.). Cells were treated with a compound for 24 h and then labeled with 10 µM EdU buffer at 37 °C for 4 h, then fixed with 4% paraformaldehyde for 30 min. The cells were incubated with glycine (2 mg/mL) for 5 min, followed by two washes with PBS containing 3% BSA. The cells were permeabilized with 0.5% Triton X-100 for 20 min and blocked with 3% bovine serum albumin (BSA). The Click-iT reaction cocktail was added and the cells were incubated in the dark for 30 min, then stained with Hoechst 33,342 solution (1:2000 dilution) for 15 min. The slides were sealed with an anti-fluorescence quencher. After image acquisition, Image J software was used to calculate the proportion of EdU-positive cells.

### Cell cycle analysis

Cells were treated with varying concentrations of a compound for 48 h, then digested with pancreatic enzymes, and fixed overnight at 4 °C in 75% ethanol. The cells were subsequently treated with 100 µg/mL RNase solution and stained with propidium iodide (PI) for 30 min. DNA content was analyzed using a BD LSRFortessa Flow Cytometer.

### Apoptosis analysis

Cells treated with varying concentrations of a compound were collected, including both floating and adherent cells, and then centrifuged. The cells were washed with PBS, followed by staining with FITC/Annexin V and propidium iodide using the Annexin Detection kit (1133534, BD Pharmingen). Apoptosis rates were measured using the Accuri C6 Flow Cytometer (BD Biosciences).

### Stable overexpression of PRMT5, KLF5 and CRBN

The full-length PRMT5, KLF5 and CRBN genes were constructed through polymerase chain reaction (PCR) and cloned into the PCDH vector. HEK-293T cells were used to package the lentiviruses. After transfection for 48 h, the lentiviruses were collected to infect HCC1806 and HCC1937 cells. Two days later, the cells were selected with 2 µg/ml puromycin. The sequences of the primers used in this study are listed in Table S3.

### siRNA transfection

All siRNAs were purchased from RIBOBIO (Guangzhou, China). Transfection was carried out using Opti-MEM and Lipofectamine™ 2000 (Invitrogen) at a final concentration of 20 nM. The siRNA sequences are listed in Table S4.

### Ubiquitination assays

The plasmids were transferred into HEK293T cells and then treated with YZ-836P for 48 h. Cells were harvested in lysis buffer (50 mM Tris-Cl, 1% SDS and 1 mM EDTA; pH 6.8) using a six-well plate. Each well contained 150 µL lysis buffer. The cell lysate was boiled for 20 min to denature proteins. 1.0 mL BSA buffer (50 mM Tris-Cl, 180 mM NaCl, 0.5% NP-40, and 0.5% BSA; pH 6.8) was added to dilute the samples. Flag-M2 beads (30 µL per sample; prewashed with BSA buffer for 3 times) were added to immunoprecipitate Flag-PRMT5 overnight with rotation in a cold room (4 °C). The second day, beads were washed 5 times with 1 mL ice-cold BSA buffer, resuspended in 60 µL of 2× SDS-PAGE sample buffer, boiled for 10 min, and centrifuged for 2 min at 12,000 g. The supernatant was subjected to Western blotting.

### In vivo tumorigenesis assays

Nude mice (approximately 6 weeks old) purchased from SJA Laboratory Animal Co., Ltd. (Changsha, China) were housed in the Specific Pathogen Free (SPF) animal facility at the Kunming Institute of Zoology, Chinese Academy of Sciences. HCC1806 cells (1 × 10^6^ cells/spot) were injected bilaterally into the 4th pair of mouse mammary gland fat pads. When the tumor volume approached 50 mm³, the mice were randomly divided into a control group and a treatment group. The treatment group received intraperitoneal injections of YZ-836P (50 mg/kg) every other day 4 times. Tumor size was measured with a vernier caliper and mouse body weight was recorded using a scale every 2 days. After 15 days, the tumors were harvested for analysis. This animal experiment followed the ARRIVE guidelines and had been approved by the Animal Ethics Committee of the Kunming Medical University (kmmu20240729).

### Patient-derived organoids

Tumor tissues were obtained from breast cancer surgery patients at the Yunnan Cancer Hospital, with informed consent from all participants and/or their legal guardians. Immediately after surgical collection, fresh breast cancer tissues were placed in DMEM/F12 medium containing 50 µg/mL of antibiotics and transported to the laboratory. The tissues were washed with sterile 1×PBS, minced with surgical scissors, and digested in a solution containing collagenase I, III, and IV (0.5-1 mg/mL), hyaluronidase (0.05–0.1 mg/mL), DMEM/F12, 10 mM HEPES, 2% BSA, 0.48 µg/mL hydrocortisone, 5 µM Y-27,632, and 50 µg/mL primocin at 37 °C for 1–2 h. After digestion, the cells were passed through a 100 µM filter, centrifuged at 500 g for 3 min, resuspended in 1×PBS, and treated with erythrocyte lysate for 3–5 min. After another wash with 1×PBS, the cells were resuspended in matrigel and seeded into a 24-well plate. They were allowed to solidify for 30 min before adding organoid culture medium. Organoid growth was monitored, and the medium was changed regularly. Once organoids reached sufficient size and quantity, they were digested, counted, and plated in a 96-well plate for treatment with YZ-836P. The viability of organoid cells after 2 days of treatment with various concentrations of YZ-836P was assessed using an ATPase activity assay kit (Promega). This study conformed the Declaration of Helsinki and had been approved by the Ethics Committee of Yunnan Cancer Hospital (KYCS2023-078).

### Statistical analysis

All statistical analyses in this study were performed using Prism v.8.0 (GraphPad). Data were presented as mean ± Standard Error of Mean (SEM). Statistical methods included two-way ANOVA or two-tailed Student’s t-test. A p-value of < 0.05 was considered statistically significant. Significance levels were indicated as * for *p* < 0.05, ** for *p* < 0.01, and *** for *p* < 0.001.

## Results

### Identification of a CRBN-recruiting PRMT5 degrader YZ-836P

**Fig. 1:**
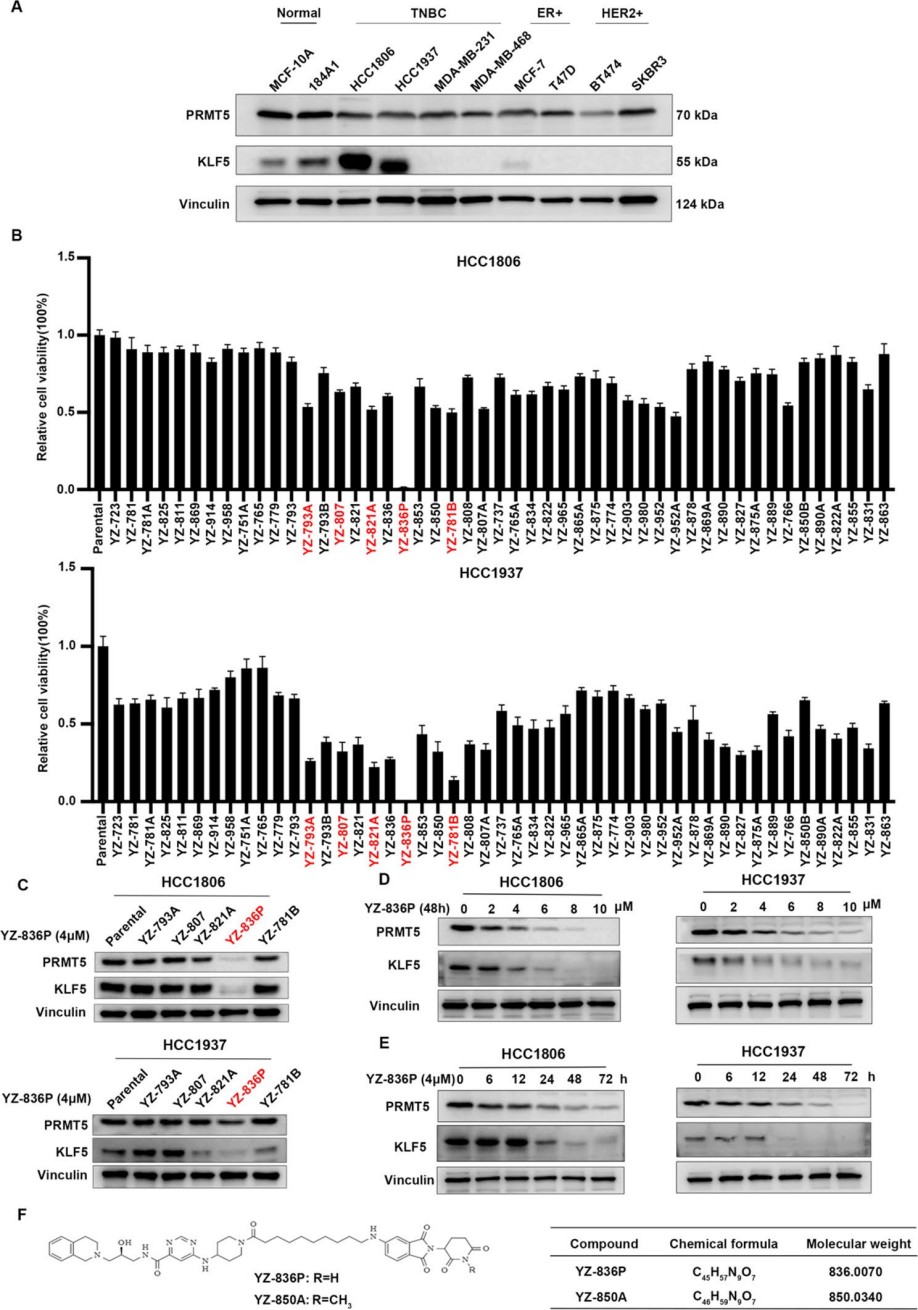
YZ-836P was identified as a PRMT5 PROTAC in TNBC. (**A**) PRMT5 and KLF5 protein expression levels in breast cancer cell lines and immortalized breast epithelial cell lines were detected by WB. (**B**) The cytotoxic effects of 49 compounds on HCC1806 and HCC1937 cells were assessed by the SRB assay (48 h, 4 µM). (**C**) Evaluation the effects of candidate compounds (48 h, 4 µM) on the protein level of PRMT5 and KLF5 by WB. (**D**) YZ-836P reduced PRMT5 and KLF5 protein levels in a concentration-dependent manner (48 h) in both HCC1806 and HCC1937 cell lines, as determined by WB. (**E**) YZ-836P could reduce PRMT5 and KLF5 proteins in a time-dependent manner (48 h, 4 µM). (**F**) The molecular formula and molecular weight of YZ-836P and YZ-850A

To design PRMT5-targeting PROTACs with killing TNBC cells in a short period of time (48–96 h), we selected GSK3326595, a PRMT5 inhibitor in Phase II clinical trials (NCT04676516), to serve as the POI binding ligand. Consisting with the binding mode of EPZ015666 and PRMT5, we concluded the piperidinyl group of GSK3326595 was solvent-exposed and the acetamide residue was a suitable position to attach a linker and E3 ligand [32, 33]. Currently, dozens of PROTACs are in clinical investigations, and most of them are CRBN-recruiting PROTACs. In addition, CRBN binding moiety perform lower molecular weight and more favorable pharmacokinetic properties [34]. For the selection of E3 ligands, considering the VHL-recruiting PROTAC MS4322 exhibits modest degradation ability, we intended to utilize more preferred E3 ligase CRBN to design PRMT5-targeting PROTAC in our research. Thus, we selected Pomalidomide and analogues as CRBN ligands to develop a series of novel PRMT5 degraders. The synthesis procedure of the PRMT5 degrader is in Figure S1. According to our earlier study, KLF5 is an important downstream target protein for PRMT5 [11]. Notably, the TNBC cell lines HCC1806 and HCC1937 show high expression levels of both PRMT5 and KLF5 (Fig. 1A). Based on this observation, we chose these two cell lines for compound screening. Through screening using the SRB assay, the compounds YZ-793A, YZ-807, YZ-821A, YZ-836P, and YZ-781B were identified to significantly reduce the cell viability of HCC1806 and HCC1937 cells after 48 h (Fig. 1B). Among these five compounds, YZ-836P showed the strongest inhibition activities. As expected, YZ-836P dramatically decreased the protein levels of PRMT5 and KLF5 (Fig. 1C). Consequently, we demonstrated that YZ-836P reduced the levels of PRMT5 and KLF5 protein, in dose- and time-dependent manners (Fig. 1D-E). Considering the characteristics of PROTAC, lower concentrations of YZ-836P had no degradation effect on PRMT5 and KLF5 (Figure S2A-B). Finally, We tested the protein degradation effect of the negative control YZ-850A and found that the degradation effect of YZ-850A were worse to YZ-836P (Figure S3A-B). The molecular formula and molecular weight of YZ-836P and negative control YZ-850A are depicted in Fig. 1F.

### YZ-836P reduces TNBC cell viability and DNA synthesis

**Fig. 2:**
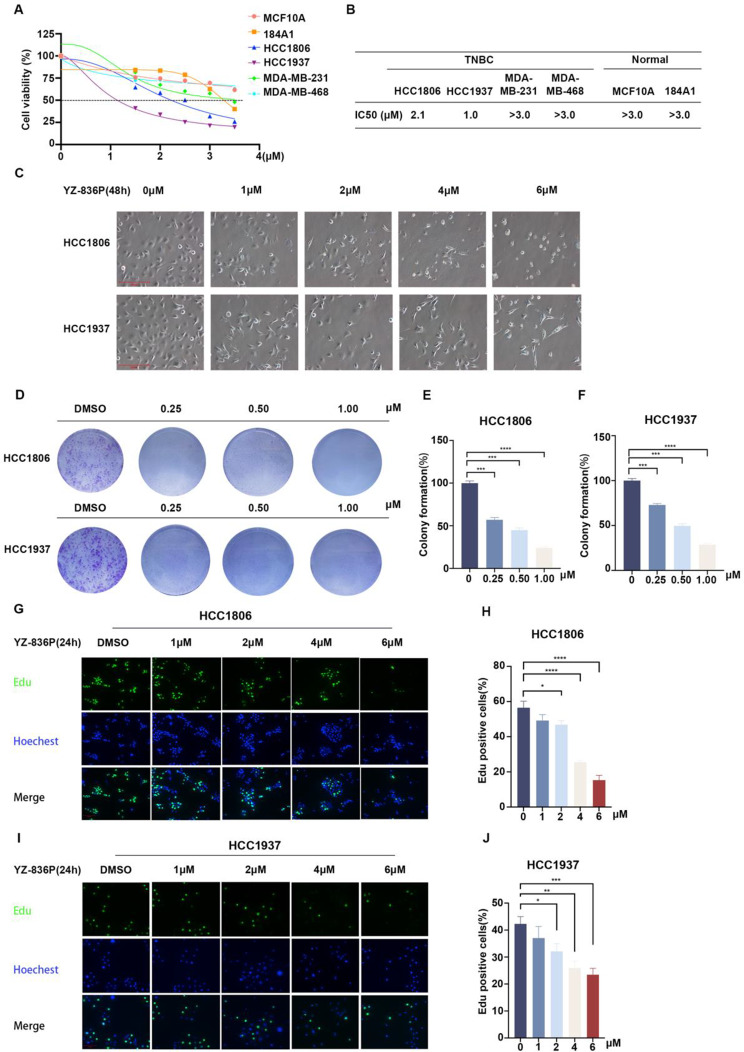
YZ-836P inhibits the growth of TNBC. (**A**) YZ-836P reduced the viability of various TNBC cell lines and immortalized breast epithelial cell lines. (**B**) Statistical analysis of the IC_50_ values of YZ-836P in six cell lines. (**C**) Microscopic observation of morphological changes of HCC1806 and HCC1937 cells after treatment with YZ-836P. (**D**) YZ-836P inhibits the colony formation of HCC1806 and HCC1937 cells. (**E**) Inhibitory effects of YZ-836P on colony formation in HCC1806 cells. The graphs showed statistical results of colony formation (*n* = 3 per group). (**F**) Inhibitory effects of YZ-836P on colony formation in HCC1937 cells. (**G**) YZ-836P inhibited DNA synthesis of HCC1806 cells. EdU incorporation assays were used to measure the effects of different concentrations of YZ-836P on DNA synthesis (24 h). Blue represented Hoechst staining, and green represented EdU staining. (**H**) Statistical results of the proportion of EdU-positive HCC1806 cells after treatment with YZ-836P (*n* = 6 per group). (**I**) YZ-836P inhibited DNA synthesis in HCC1937 cells. (**J**) Statistical results of the proportion of EdU-positive HCC1937 cells after treatment with YZ-836P (*n* = 6 per group). Data represent results from three independent experiments. *p < 0.05, **p < 0.01, and ***p < 0.001; ns, not significant

Subsequently, we measured the half-maximal inhibitory concentration (IC_50_) of YZ-836P in two immortalized breast epithelial cell lines, MCF10A and 184A1, and four TNBC cell lines. In HCC1806 and HCC1937 cells, YZ-836P had marginally cytotoxic effects, with IC_50_ values of 2.1 µM and 1.0 µM, which were significantly superior to those of YZ-850A. (Fig. 2A-B and Figure S3C). IC_50_ values of YZ-836P in other cell lines are larger than 3.0 µM. Therefore, HCC1806 and HCC1937 cells were chosen for additional research. YZ-836P induced nuclear fragmentation, cellular shrinkage, and cell separation from neighboring cells (Fig. 2C). Consistently, YZ-836P inhibited the formation of colonies in a concentration-dependent manner (Fig. 2D-F). Additionally, YZ-836P inhibited DNA synthesis of HCC1806 and HCC1937 cells in a concentration-dependent manner (Fig. 2G-J).

### YZ-836P induces cell cycle arrest and apoptosis in TNBC cells

**Fig. 3:**
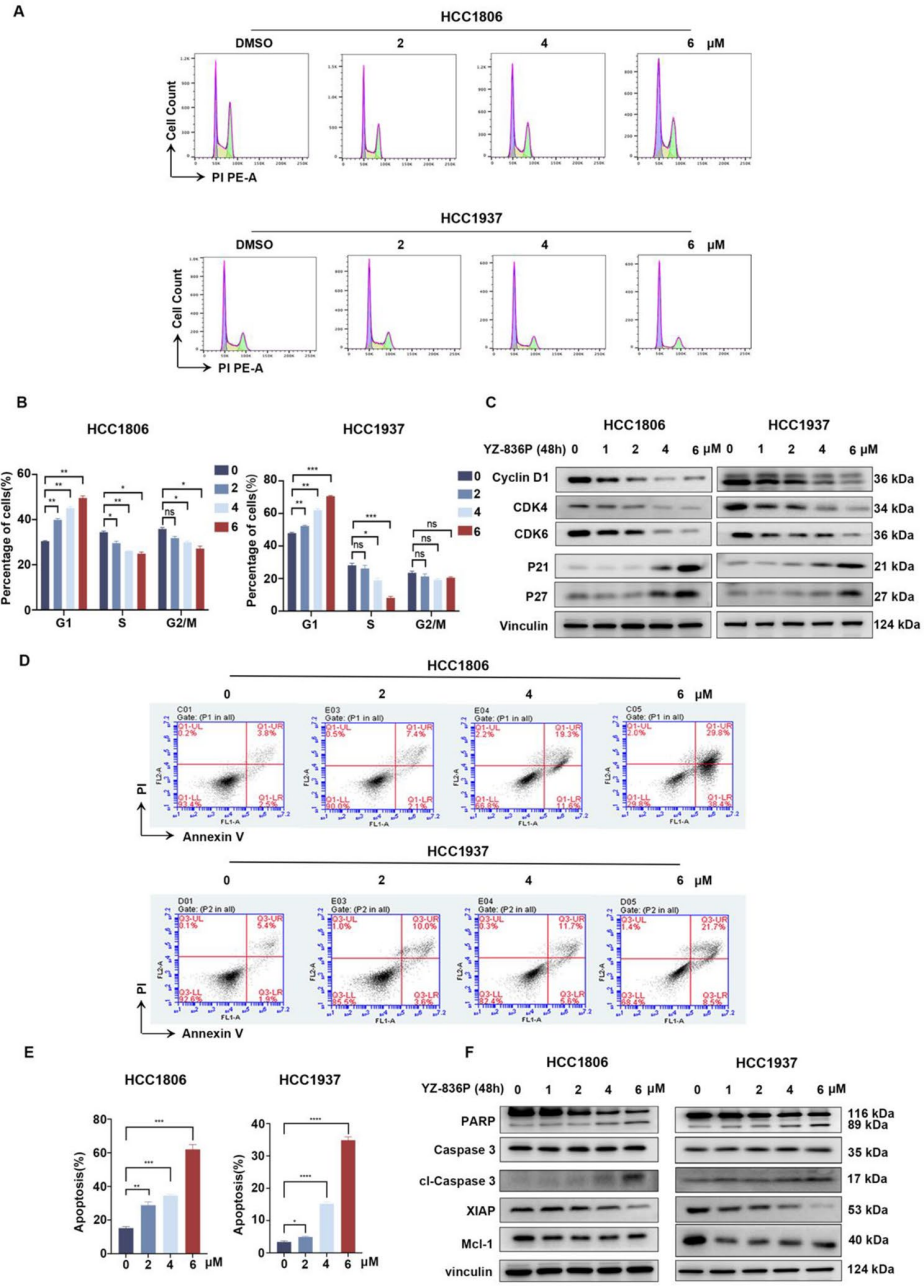
YZ-836P induces cell cycle arrest and promotes apoptosis in TNBC. (**A**) YZ-836P increased the proportion of cells in the G1 phase. HCC1806 and HCC1937 cells were incubated with YZ-836P, stained with PI, and analyzed using flow cytometry (48 h). (**B**) YZ-836P induced the G1 phase cell cycle arrest in both cell lines. (**C**) YZ-836P regulated the expression levels of cell cycle-related proteins, including Cyclin D1, CDK6, CDK4, p21, and p27, as detected by WB. (**D**) YZ-836P induces apoptosis in both cell lines, as measured by Annexin V-PI double staining and flow cytometry. (**E**) Statistical results of panel D. (**F**) YZ-836P regulated the expression of apoptosis-related proteins, including cleaved Caspase 3 and PARP, XIAP, and Mcl-1, as detected by WB. Data represent results from three independent experiments. *p < 0.05, **p < 0.01, and ***p < 0.001; ns, not significant

We sought to test whether YZ-836P induces cell cycle arrest. After 48 h treatment of YZ-836P, we used flow cytometry to examine HCC1806 and HCC1937 cell cycle distributions. We found that there was a concentration-dependent increase of the G1 phase population and a decrease of S phase cells (Fig. 3A-B). Consistently, the expression of cell cycle-related proteins CDK4, CDK6, and Cyclin D1 was decreased by YZ-836P in both cell lines in a dose-dependent manner. At the same time, YZ-836P caused a significant expression upregulation of cell cycle kinase inhibitors p21 and p27 (Fig. 3C). We further explored whether YZ-836P induced TNBC apoptosis. As expected, YZ-836P significantly increased the percentage of Annexin-V positive cells (Fig. 3D-E). Additionally, YZ-836P promoted a dose-dependent increase of cleaved PARP and Caspase 3, suggesting that YZ-836P induces apoptosis through the activation of the mitochondrial-dependent pathway. This was further supported by the decrease in the levels of the anti-apoptotic proteins XIAP and Mcl-1 (Fig. 3F).

### YZ-836P inhibits TNBC via the PRMT5-KLF5 pathway

**Fig. 4:**
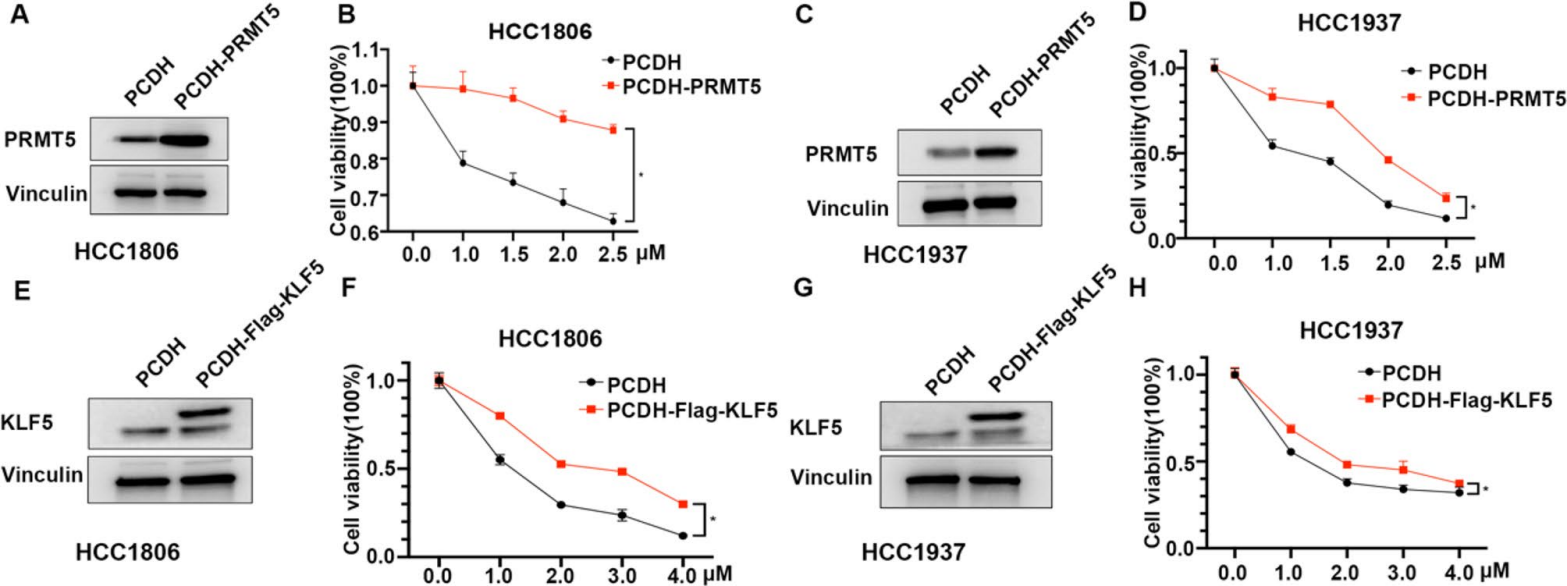
YZ-836P inhibits TNBC via the PRMT5-KLF5 pathway. (**A**) PRMT5 overexpression in HCC1806 cells, as demonstrated by WB. (**B**) PRMT5 overexpression in HCC1806 cells partially and significantly blocked the cytotoxic effects of YZ-836P (48 h). (**C**) PRMT5 overexpression in HCC1937 cells, as demonstrated by WB. (**D**) PRMT5 overexpression in HCC1806 cells partially and significantly blocked the cytotoxic effects of YZ-836P (48 h). (**E**) KLF5 overexpression in HCC1806 cells, as demonstrated by WB. (**F**) KLF5 overexpression in HCC1806 cells partially and significantly blocked the cytotoxic effects of YZ-836P (48 h). (**G**) KLF5 overexpression in HCC1937 cells, as demonstrated by WB. (**H**) KLF5 overexpression in HCC1806 cells partially and significantly blocked the cytotoxic effects of YZ-836P (48 h). Data represent results from three independent experiments. *p < 0.05, **p < 0.01, and ***p < 0.001; ns, not significant

To investigate whether YZ-836P inhibits TNBC through targeting PRMT5 for degradation, we constructed stable PRMT5 overexpression HCC1806 and HCC1937 cell lines. As expected, the cytotoxic effects of YZ-836P on TNBC cells were significantly blocked by overexpressing PRMT5 (Fig. 4A-D). Since PRMT5 functions predominately through stabilization of KLF5 in TNBC [11], we wondered whether YZ-836P inhibits TNBC also through KLF5. Therefore, we also constructed KLF5 overexpression HCC1806 and HCC1937 cell lines and demonstrated that KLF5 overexpression similarly attenuated the cytotoxic effects of YZ-836P on TNBC cells (Fig. 4E-H). These results suggest that YZ-836P’s inhibitory effects on TNBC cells are mediated, at least partially, through the degradation of PRMT5 and the subsequent destabilization of KLF5.

### YZ-836P interacts with and degrades PRMT5 via the ubiquitin-proteasome system

**Fig. 5:**
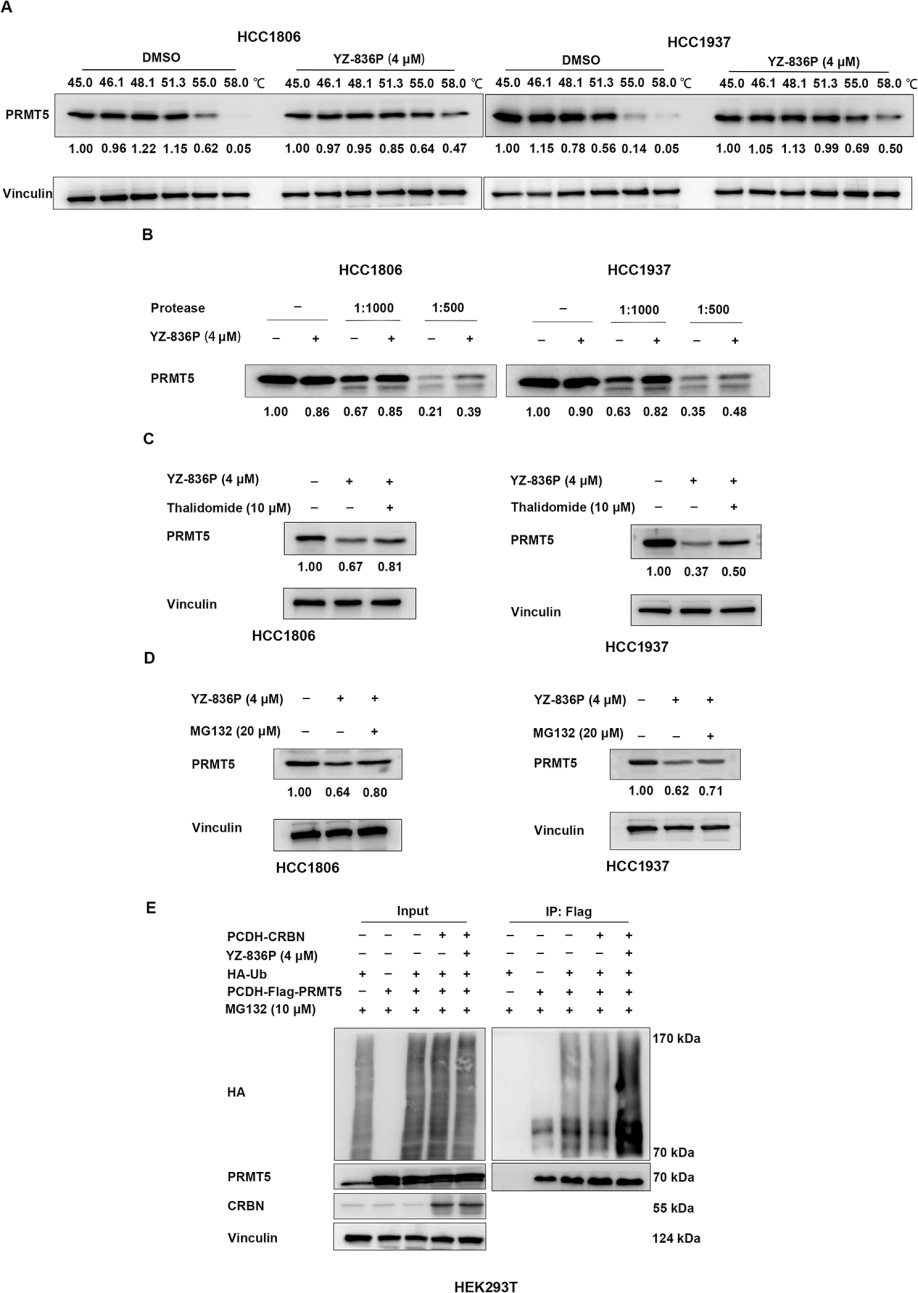
YZ-836P interacts with and degrades PRMT5 via the CRBN-mediated ubiquitination and proteasomal degradation. (**A**) CETSA assays revealed that YZ-836P increased the thermostability of PRMT5 protein. (**B**) DARTS assays revealed that YZ-836P decreased the degradation of PRMT5 protein by proteases. (**C**) Thalidomide (10 µM) treatment reversed YZ-836P-induced degradation of PRMT5 protein in HCC1806 cells (48 h) and HCC1937 cells (48 h). (**D**) MG132 (20 µM) treatment reversed YZ-836P-induced degradation of PRMT5 protein in HCC1806 cells (48 h) and HCC1937 cells (48 h). (**E**) YZ-836P increased ubiquitination of PRMT5 in HEK293T cells. Data represent results from three independent experiments. *p < 0.05, **p < 0.01, and ***p < 0.001; ns, not significant

To test whether YZ-836P interacts with PRMT5, we tested the thermostability of the PRMT5 protein through a cellular thermal shift assay (CETSA). In agreement with our hypothesis, within the same temperature gradient, the degradation rate of PRMT5 protein was decreased by YZ-836P when compared to the DMSO control group (Fig. 5A). Next, a drug affinity responsive target stability assay (DARTS) was also carried out. This assay is based on the principle that target proteins, once bound to a specific compound, become less susceptible to protease degradation [35]. The results indicated that, upon addition of an equal proportion of protease, the protein degradation rate was significantly decreased by YZ-836P (Fig. 5B).

YZ-836P, as a PROTAC molecule, should bind to CRBN, inducing the ubiquitination and degradation of PRMT5. Therefore, CRBN should play a crucial role in YZ-836P-induced degradation of PRMT5. It has been documented that thalidomide is capable of forming a complex with CRBN, affecting CRBN-mediated substrate recruiting. Firstly, we employed siRNA to knockdown CRBN in HCC1806 and HCC1937 cells and found the cytotoxic effects of YZ-836P on TNBC cells were significantly blocked (Figure S4A-D). As expected, thalidomide retarded the YZ-836P-induced degradation of PRMT5 (Fig. 5C). Additionally, the proteasome inhibitor MG132 blocked the YZ-836P-induced degradation of PRMT5 (Fig. 5D). Finally, we demonstrated that YZ-836P significantly increased the ubiquitination level of PRMT5 in HEK293T cells (Fig. 5E). These results suggest that YZ-836P recruits CRBN to induce the ubiquitination and proteasomal degradation of PRMT5.

### YZ-836P inhibits growth of TNBC patient-derived organoids and xenograft tumors in vivo

**Fig. 6:**
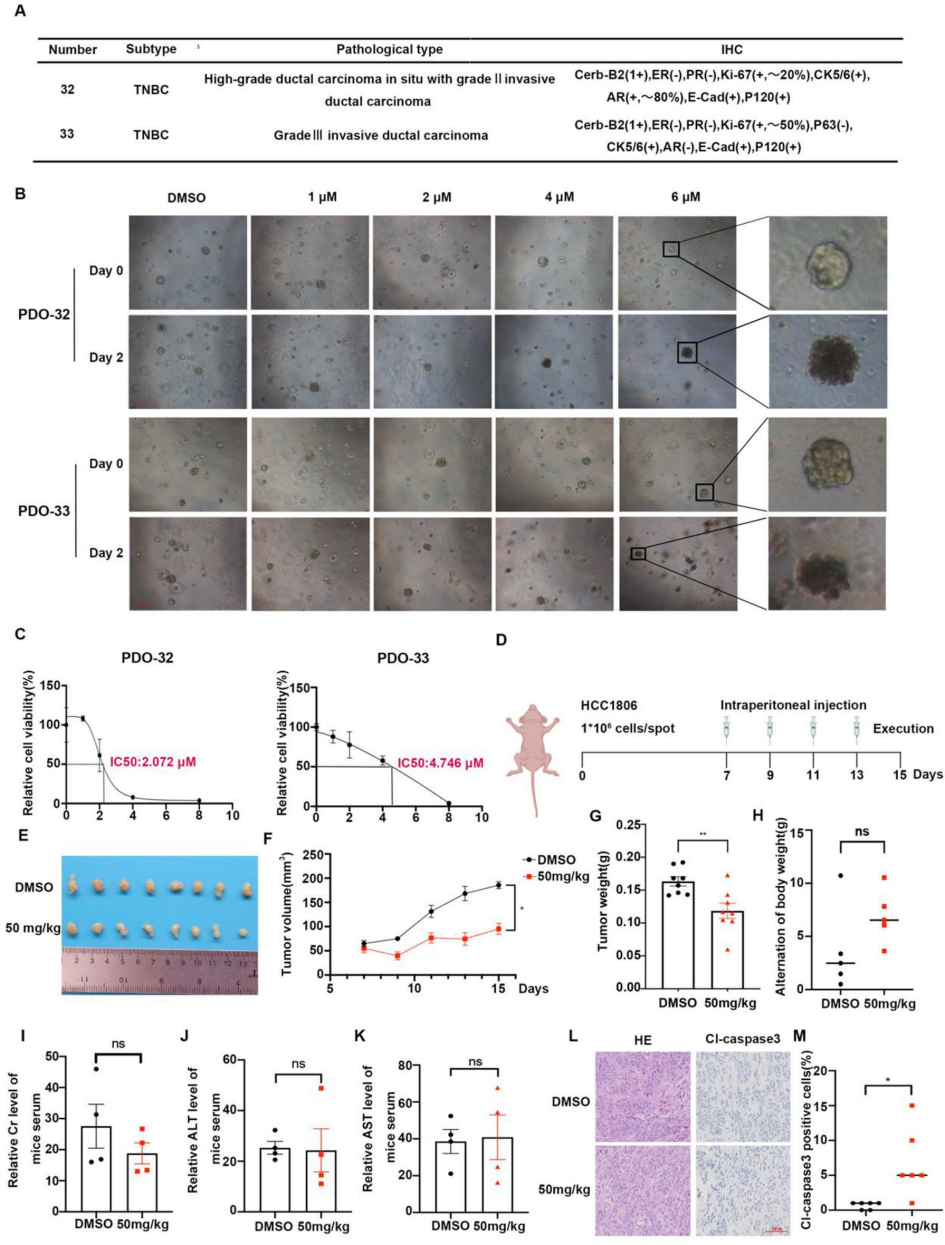
YZ-836P inhibits growth of TNBC patient-derived organoids and xenograft tumors in vivo. (**A**) Pathological data of two TNBC patients. (**B**) YZ-836P disrupted the structural integrity of PDOs. Microscopic observation of PDOs morphology before and after 48 h of YZ-836P treatment. Arrows indicated decomposing PDOs. (**C**) YZ-836P inhibited ATP activity in PDOs. Cell viability changes in PDOs treated with YZ-836P for 48 h, measured by using an ATPase activity assay kit. (**D**) Schematic of the xenograft tumor model in nude mice and treatment protocol. (**E**) YZ-836P inhibited xenograft tumor growth. Mice were sacrificed 15 days after tumor inoculation, and tumors were collected and photographed (*n* = 8 per group). (**F**) YZ-836P reduced xenograft tumor volume. Tumor volume was measured every other day starting from the 7^th^ day after inoculation (*n* = 8 per group). (**G**) YZ-836P decreased xenograft tumor weight. Tumor weight was measured after collection (*n* = 8 per group). (**H**) Body weight changes in nude mice before and after YZ-836P treatment (*n* = 4 per group). (**I**) YZ-836P treatment did not significantly affect serum creatinine (Cr) levels (*n* = 4 per group). (**J**) YZ-836P treatment did not significantly affect serum alanine aminotransferase (ALT) levels (*n* = 4 per group). (K) YZ-836P treatment did not significantly affect serum aspartate aminotransferase (AST) levels (*n* = 4 per group). (**L**) Immunohistochemical analysis of cl-Caspase 3 expression in xenograft tumors, with representative images. (**M**) YZ-836P treatment significantly increased cl-Caspase 3 expression in xenograft tumors (*n* = 6 per group). Data represent results from three independent experiments. *p < 0.05, **p < 0.01, and ***p < 0.001; ns, not significant

Patient-derived organoids (PDOs), cultured in vitro in a 3D environment, accurately replicate the architecture and maintain the heterogeneity of primary tumors, making them valuable tools for drug efficacy evaluations [36]. We sourced tumor specimens from two patients diagnosed with the TNBC to create two PDO models (Fig. 6A). These PDOs were treated with different concentrations of YZ-836P. Microscopic observation revealed that the DMSO control group preserved their coherent and organized structure, while the YZ-836P treated group fragmented into smaller aggregates and displayed signs of necrosis. Furthermore, ATPase activity assays revealed a notable reduction in cell viability upon YZ-836P treatment, with PDO #33 showing a higher IC50 compared to PDO #32, a discrepancy potentially rooted in the original tumor’s greater malignancy in PDO #33 (Fig. 6B-C).

To assess YZ-836P’s anti-tumor efficacy in vivo, we inoculated HCC1806 cells into the 4^th^ pair of mammary gland fat pads of immunodeficient mice. Once the xenografts reached 50 mm³, the mice were randomly grouped and treated with YZ-836P by intraperitoneal injections. The treated group showed significantly smaller tumor volumes and weights compared to the control group, demonstrating the in vivo anti-tumor efficacy of YZ-836P (Fig. 6D-G). Notably, there were no discernible effects on the mouse’s body weights (Fig. 6H), or serum levels of alanine transaminase (ALT), aspartate transaminase (AST), and creatinine (Cr), suggesting a tolerable safety profile for YZ-836P (Fig. 6I-K). Moreover, immunohistochemical analysis of paraffin-embedded tumor specimens from the mice showed a significant increase in the proportion of cleaved Caspase 3 positive cells, indicating enhanced apoptotic activity following YZ-836P treatment (Fig. 6L-M).

## Discussion

PRMT5, a type II protein arginine methyltransferase, is highly expressed in various solid tumors and hematologic malignancies [37, 38]. Multiple studies have shown that PRMT5 expression is correlated with overall survival and is associated with tumor metastasis [39, 40]. Therefore, developing effective PRMT5 inhibitors is crucial for improving cancer treatment. GSK3326595 is currently the most advanced PRMT5 small-molecule inhibitor in development. However, further research revealed that GSK3326595, designed based on the *S*-adenosylmethionine (SAM) substrate, has significant toxicity, with 89% of participants experienced adverse events, including thrombocytopenia, anemia, and fatigue, may hinder its clinical application [41]. Then, we synthesized a series of novel PROTAC compounds and identified YZ-836P as a lead compound with superior tumor inhibitory effects. YZ-836P effectively inhibited the viability and DNA synthesis of TNBC cells (Fig. 2D-J), induced G1 phase cell cycle arrest (Fig. 3A-B), triggered apoptosis by activating the Caspase 3 cascade and inhibiting the expression of anti-apoptotic proteins (Fig. 3D-F) and inhibited the growth of xenograft tumors in nude mice (Fig. 6E-F). Additionally, YZ-836P has a tolerable safety profile, (Fig. 6G), with no significant toxicity in vivo (Fig. 6H-K). These results indicate that YZ-836P exhibits potent anti-tumor activity and could be a promising therapeutic agent for the treatment of TNBC.

Innovatively, we conducted an in vitro drug sensitivity evaluation of YZ-836P using the PDO model. PDOs are derived directly from patient tumor tissues and contain cancer stem cells from the primary tumor. These cancer stem cells interact with other cell types, forming a complex microenvironment that closely mimics the in vivo state of the primary tumor [42–44]. As substitutes for animal and traditional in vitro models, PDOs provide a superior research platform for exploring the molecular mechanisms of disease development and personalized treatment [45–48]. Our findings indicate that YZ-836P treatment disrupted the integrity of TNBC PDOs and inhibited their cell viability (Fig. 6B-C).

YZ-836P effectively degrades PRMT5 in TNBC cell lines HCC1806 and HCC1937 (Fig. 1D-E), showing a more pronounced effect compared to previously reported PRMT5 PROTAC compound MS4322 [32]. In MCF-7 cells, MS4322 at 5 µM initiated PRMT5 degradation on day 2, peaking by day 8, while inhibiting cell growth by approximately 50% after 6 days at concentrations of 3 µM and 10 µM. In contrast, YZ-836P achieved PRMT5 degradation within 6 h at 4 µM, reaching optimal degradation in 2 to 3 days (Fig. 1E). We further investigated YZ-836P’s effect on the proliferation of TNBC cell lines and found that after 2 days of treatment, YZ-836P inhibited the growth of HCC1806 and HCC1937 cells by 50% at concentrations of 2.1 µM and 1.0 µM (Fig. 2A-B). Furthermore, overexpression of PRMT5 at the cellular level partially reversed the cytotoxic effects of YZ-836P on TNBC cells (Fig. 4A-D). CETSA assay indicated that the degradation rate of PRMT5 protein slowed in the YZ-836P treatment group, and PRMT5 protein exhibited higher thermal stability when bound to YZ-836P (Fig. 5A). Similarly, upon the addition of an equal proportion of protease, the protein degradation rate in the YZ-836P treated groups slowed down (Fig. 5B).

PRMT5 regulates the transcription of target genes and influences downstream biological processes by methylating substrate proteins, such as KLF5, c-Myc, Slug, Cyclin D1 [11, 49, 50]. Our previous research found that overexpressing KLF5 can partially reverse the cell growth arrest and reduced DNA synthesis caused by PRMT5 knockdown [11]. In this study, when we overexpressed KLF5 in TNBC cells, we noted a partial mitigation of YZ-836P-induced cytotoxic effects, including cell growth inhibition and enhanced apoptosis (Fig. 4E-H). This finding highlights the importance of the PRMT5/KLF5 pathway in mediating the anti-tumor effects of YZ-836P in TNBC. Given that KLF5 is a downstream effector of PRMT5, with its transcriptional activity tightly controlled by PRMT5-mediated methylation, it stands to reason that YZ-836P, by inhibiting PRMT5 methyltransferase activity, diminishes the methylation status of KLF5. This, in turn, impairs KLF5’s ability to promote cell proliferation and suppress apoptosis.

Despite the significant potential of PROTAC technology in drug development, it still faces several limitations and challenges [51, 52]. The unique structure of PROTAC molecules results in a relatively high molecular weight, usually above 700. In this study, the molecular weight of YZ-836P is 836.007 (Fig. 1F). This can restrict its application in certain drug delivery systems and may affect its pharmacokinetic properties *in vivo.* Although PROTACs are designed to specifically degrade target proteins, non-specific effects on other proteins can still occur in practical applications. The YZ-836P also has the possibility of missing the target. There are many proteins that are similar in structure and function. If the target protein is structurally highly like to other proteins, the PROTAC molecule may incorrectly recognize and degrade these similar proteins, resulting in an off-target effect. The drug concentration and kinetic properties of PROTAC molecules, complex internal environment in cells and so on may affect its off-target. However, off-target effects do not necessarily impact drug efficacy, as is the case with many kinase inhibitors that exhibit multi-target effects. These effects do not hinder their potential as anti-tumor drugs, such as EGFR inhibitors. Additionally, some proteins may lack accessible binding sites or enzymatic activity sites, making them insensitive to PROTAC technology, which complicates the development of PROTAC molecules targeting these proteins. To address these challenges and limitations, researchers are optimizing the design of PROTAC molecules, enhancing their selectivity and activity towards targets, reducing their molecular weight, and improving drug delivery systems. These efforts aim to refine PROTAC technology and expand its application in clinical treatments.

## Conclusions

Through our in-depth research, the innovative PROTAC compound YZ-836P has demonstrated exceptional anti-tumor potential in both in vitro and in vivo experiments. Its unique capability lies in its precise targeting and degradation of the PRMT5 protein, effectively killing TNBC cells via the PRMT5-KLF5 signaling pathway. The interaction between YZ-836P and CRBN facilitates the ubiquitination of PRMT5, leading to its degradation through the proteasome system. This discovery not only provides a solid theoretical foundation and experimental evidence for the development of PROTAC drugs targeting PRMT5 and similar proteins but also broadens new avenues for cancer treatment. Furthermore, the significant potential of YZ-836P in treating TNBC strongly supports its candidacy as a therapeutic agent for this challenging disease.

## Electronic supplementary material

Below is the link to the electronic supplementary material.

Supplementary Material 1

## Data Availability

The datasets used and/or analyzed during the current study are available from the corresponding author on reasonable request.

## Abbreviations

TNBC: Triple-negative breast cancer
CRBN: Cereblon
ERα: Estrogen receptor
PR: Progesterone receptor
HER2: Human epidermal growth factor receptor 2
PRMT5: Protein arginine methyltransferase 5
PROTAC: Proteolysis Targeting Chimeras
UPS: Ubiquitin-proteasome system
POI: Protein of interest
PDO: Patient-derived organoid
ALT: Alanine transaminase
AST: Aspartate transaminase
Cr: Creatinine
SAM: S-Adenosylmethionine
