# Supplementary material for: Targeting PRMT5 through PROTAC for the treatment of triple-negative breast cancer
Source: J Exp Clin Cancer Res. 2024 Nov 30;43:314. doi: 10.1186/s13046-024-03237-y (PMC11607928; doi:10.1186/s13046-024-03237-y)
Supplement: Supplementary file 1 — Supplementary Material 1 [file 13046_2024_3237_MOESM1_ESM.doc]

**Supplementary Material**

**Figure S1. Synthesis scheme of compound YZ-836P and YZ-850A**


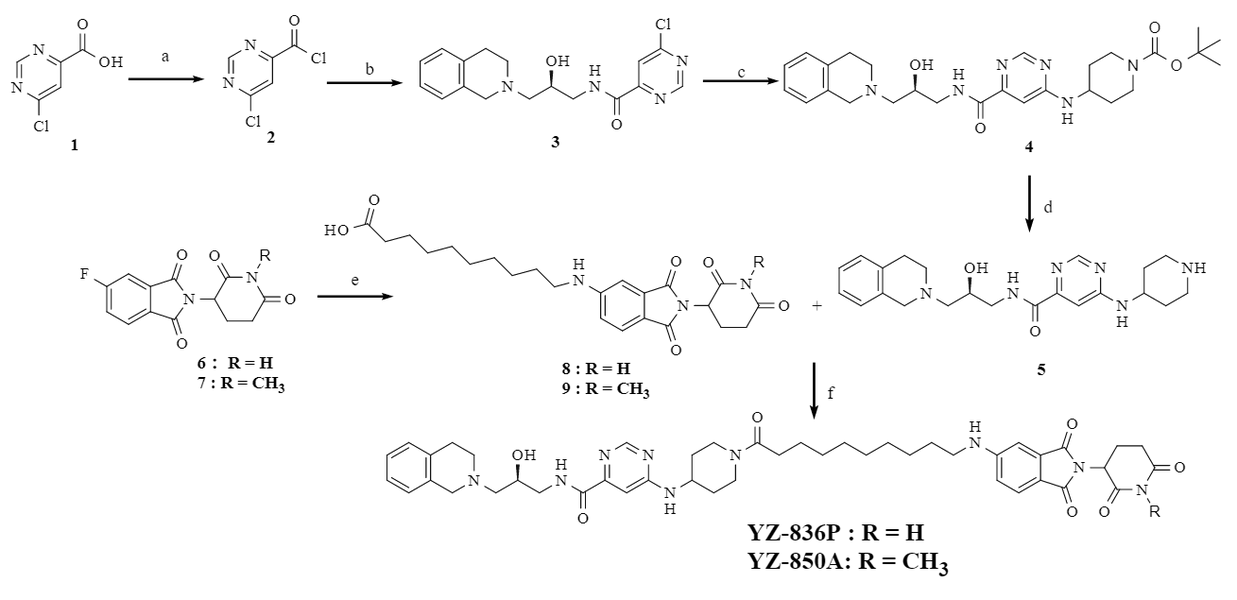


Reagents and conditions: (a) Oxalyl chloride, DMF, DCM, 40 ℃; (b) (*S*)-1-amino-3-(3,4-dihydroisoquinolin-2(1*H*)-yl)propan-2-ol, triethylamine, DCM; (c) *tert*-butyl 4-aminopiperidine-1-carboxylate, triethylamine, *i*-PrOH, 80 ℃; (d) TFA, DCM; (e) 10-aminodecanoic acid, DIEA, DMSO, N_2_, 90 ℃; (f) HATU, DIEA, DMSO.

*N*-((*S*)-3-(3,4-dihydroisoquinolin-2(1*H*)-yl)-2-hydroxypropyl)-6-((1-(10-((2-(2,6-dioxo-3-piperidinyl)-1,3-dioxo-5-isoindolinyl)amino)decanoyl)-4-piperidinyl)amino)pyrimidine-4-carboxamide (YZ-836P)

To a solution of **1** (12.36 mmol) in DCM was added Oxalyl chloride (4.6 mL). And the mixture was heated at 40 ℃. Subsequently, the DMF (0.3 mL) was added by syringe, and the reaction mixture was stirred for 5 h. The solvent was removed under vacuum to give intermediate **2**, and the intermediate **2** was used to the next step without further purification.

To a mixture of intermediate **2** (12.36 mmol) and (*S*)-1-amino-3-(3,4-dihydroisoquinolin-2(1*H*)-yl)propan-2-ol (12.36 mmol) in DCM, the triethylamine (37.08 mmol) was added slowly at room temperature for 3 h. And then the reaction mixture was concentrated under vacuum. The residue was purified by silica gel flash column chromatography to afford the intermediate **3** (60.54% yield).

To a mixture of intermediate **3** (7.49 mmol) and *tert*-butyl 4-aminopiperidine-1-carboxylate (14.97 mmol) in *i*-PrOH, and the triethylamine (14.97 mmol) was added into the solution. The reaction mixture was stirred at 80 ℃ overnight and then concentrated under vacuum. The residue was purified by silica gel flash column chromatography to afford the intermediate **4** (98.80% yield).

The intermediate **4** (7.03 mmol) was added to DCM, and the TFA (3 mL) was added to the solution. Then, the mixture was reacted at room temperature overnight. Subsequently, the solvent was removed under vacuum to give the intermediate **5**, and the intermediate **5** was used to the next step without further purification.

DIEA (2.90 mmol) was added to a solution of **6** (1.45 mmol) and 10-aminodecanoic acid (1.74 mmol) in DMSO. The mixture was degassed and charged 3 times with N_2_. Then the reaction mixture was stirred at 90 ℃ overnight. The resulting mixture was extracted with EA, and the organic layers were washed with brine, dried over anhydrous Na_2_SO_4_, and concentrated under reduced pressure. The crude residue was purified by column chromatography to give intermediate **8** (29.55% yield).

To a solution of intermediate **5** (0.43 mmol), intermediate **8** (0.43 mmol) and HATU (0.56 mmol) in DMSO, the DIEA (1.29 mmol) was added into the solution. The reaction mixture was stirred at room temperature for 3 h. The resulting mixture was diluted with H_2_O and extracted with EA, and the combined organic extracts were dried over anhydrous Na_2_SO_4_ and evaporated in vacuo. The crude residue was purified by column chromatography to afford **YZ-836P** (23.92% yield). ^1^H NMR (500 MHz, DMSO-*d*_6_) δ 11.05 (s, 1H), 8.77 – 8.73 (m, 1H), 8.29 (d, *J* = 1.2 Hz, 1H), 7.75 (d, *J* = 7.4 Hz, 1H), 7.55 (d, *J* = 8.4 Hz, 1H), 7.11 – 7.07 (m, 4H), 7.05 (d, *J* = 1.2 Hz, 1H), 7.01 (d, *J* = 6.4 Hz, 1H), 6.93 (d, *J* = 2.1 Hz, 1H), 6.85 – 6.82 (m, 1H), 5.02 (dd, *J* = 12.7, 5.4 Hz, 1H), 4.96 (d, *J* = 4.7 Hz, 1H), 4.23 (d, *J* = 13.1 Hz, 1H), 4.12 – 4.06 (m, 2H), 3.91 – 3.86 (m, 1H), 3.82 (d, *J* = 13.5 Hz, 1H), 3.65 – 3.57 (m, 2H), 3.46 – 3.39 (m, 1H), 3.17 (d, *J* = 5.2 Hz, 2H), 3.14 (d, *J* = 5.9 Hz, 2H), 2.84 – 2.81 (m, 2H), 2.79 – 2.72 (m, 2H), 2.71 – 2.66 (m, 1H), 2.29 (t, *J* = 7.4 Hz, 2H), 2.03 – 1.95 (m, 1H), 1.95 – 1.83 (m, 2H), 1.60 – 1.53 (m, 2H), 1.51 – 1.45 (m, 2H), 1.39 – 1.21 (m, 16H); HRMS (ESI): calcd for C_45_H_58_N_9_O_7_ [M+H]^+^: 836.4454, found 836.4456; HPLC purity: 98.65%, R_t_ = 8.846 min.

*N*-((*S*)-3-(3,4-dihydroisoquinolin-2(1*H*)-yl)-2-hydroxypropyl)-6-((1-(10-((2-(1-methyl-2,6-dioxopiperidin-3-yl)-1,3-dioxoisoindolin-5-yl)amino)decanoyl)piperidin-4-yl)amino)pyrimidine-4-carboxamide (YZ-850A)

The synthesized assay and condition of YZ-850A were similar to YZ-836P, only replacing compound 6 to compound 7. The crude residue was purified by column chromatography to afford **YZ-850A**. 1H NMR (500 MHz, DMSO-*d6*) δ 8.77 – 8.73 (m, 1H), 8.29 (s, 1H), 7.75 (d, *J* = 7.4 Hz, 1H), 7.55 (d, *J* = 8.3 Hz, 1H), 7.12 – 7.08 (m, 4H), 7.05 (d, *J* = 1.2 Hz, 1H), 7.01 (d, *J* = 6.5 Hz, 1H), 6.94 (d, *J* = 2.2 Hz, 1H), 6.86 – 6.82 (m, 1H), 5.12 – 5.07 (m, 1H), 4.97 (s, 1H), 4.23 (d, *J* = 12.9 Hz, 1H), 4.09 (s, 1H), 3.89 (s, 1H), 3.82 (d, *J* = 13.3 Hz, 1H), 3.62 (d, *J* = 7.9 Hz, 2H), 3.45 – 3.39 (m, 1H), 3.18 – 3.12 (m, 3H), 3.00 (s, 3H), 2.82 (d, *J* = 6.0 Hz, 2H), 2.80 – 2.71 (m, 3H), 2.32 – 2.27 (m, 2H), 2.03 – 1.98 (m, 1H), 1.91 (s, 2H), 1.59 – 1.55 (m, 2H), 1.48 (s, 2H), 1.39 – 1.22 (m, 18H); HRMS (ESI): calcd for C_46_H_60_N_9_O_7_ [M+H]^+^: 850.4610, found 850.4613.

**Figure S2. Lower concentrations of YZ-836P are unable to degrade PRMT5 and KLF5**

**
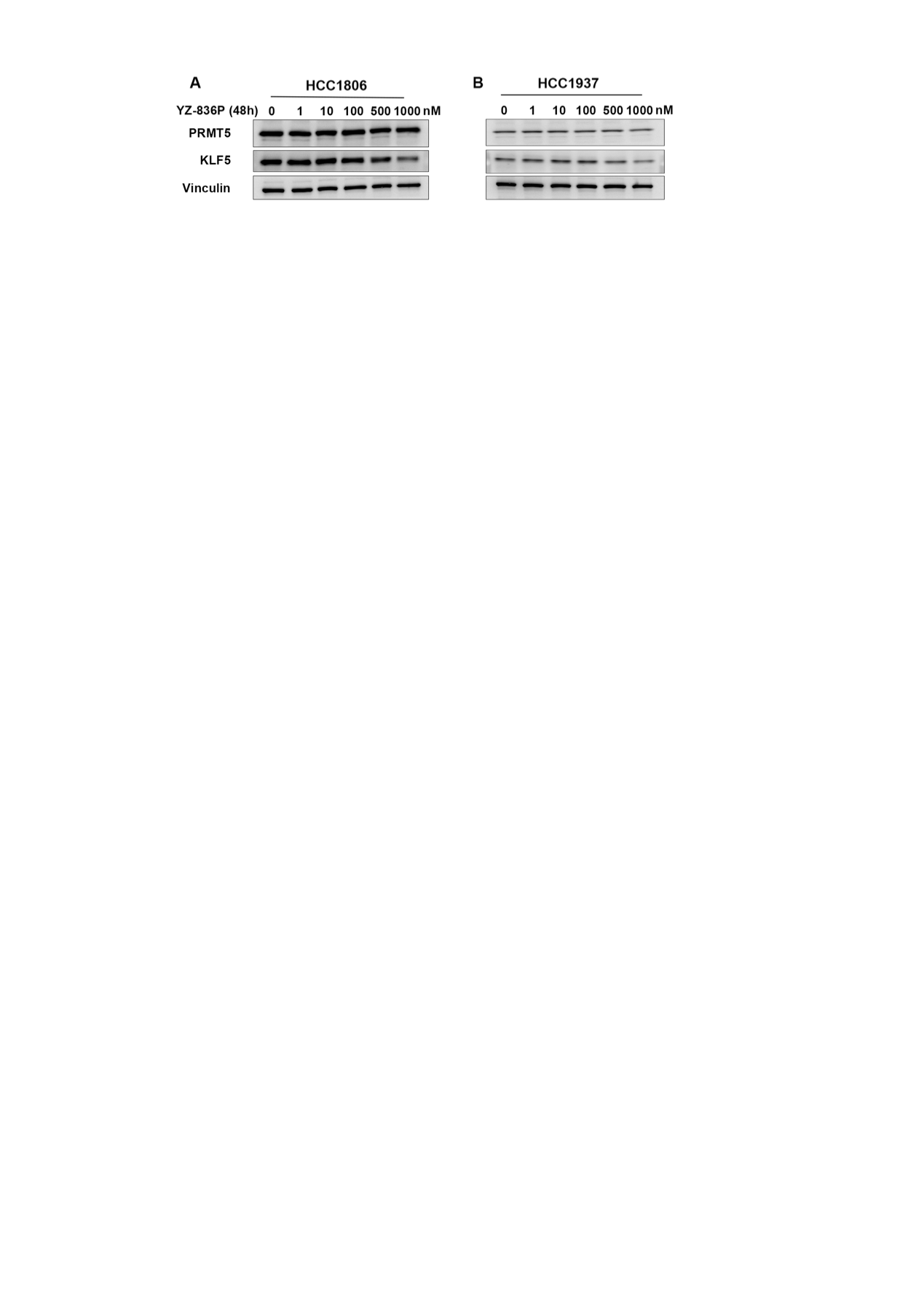
**

(A) Lower concentrations of YZ-836P could not reduce PRMT5 and KLF5 protein levels in HCC1806 cell line, as determined by WB.

(B) Lower concentrations of YZ-836P could not reduce PRMT5 and KLF5 protein levels in HCC1937 cell line, as determined by WB.

**Figure S3. The effects of the negative control YZ-850A is significantly worse than YZ-836P**

**
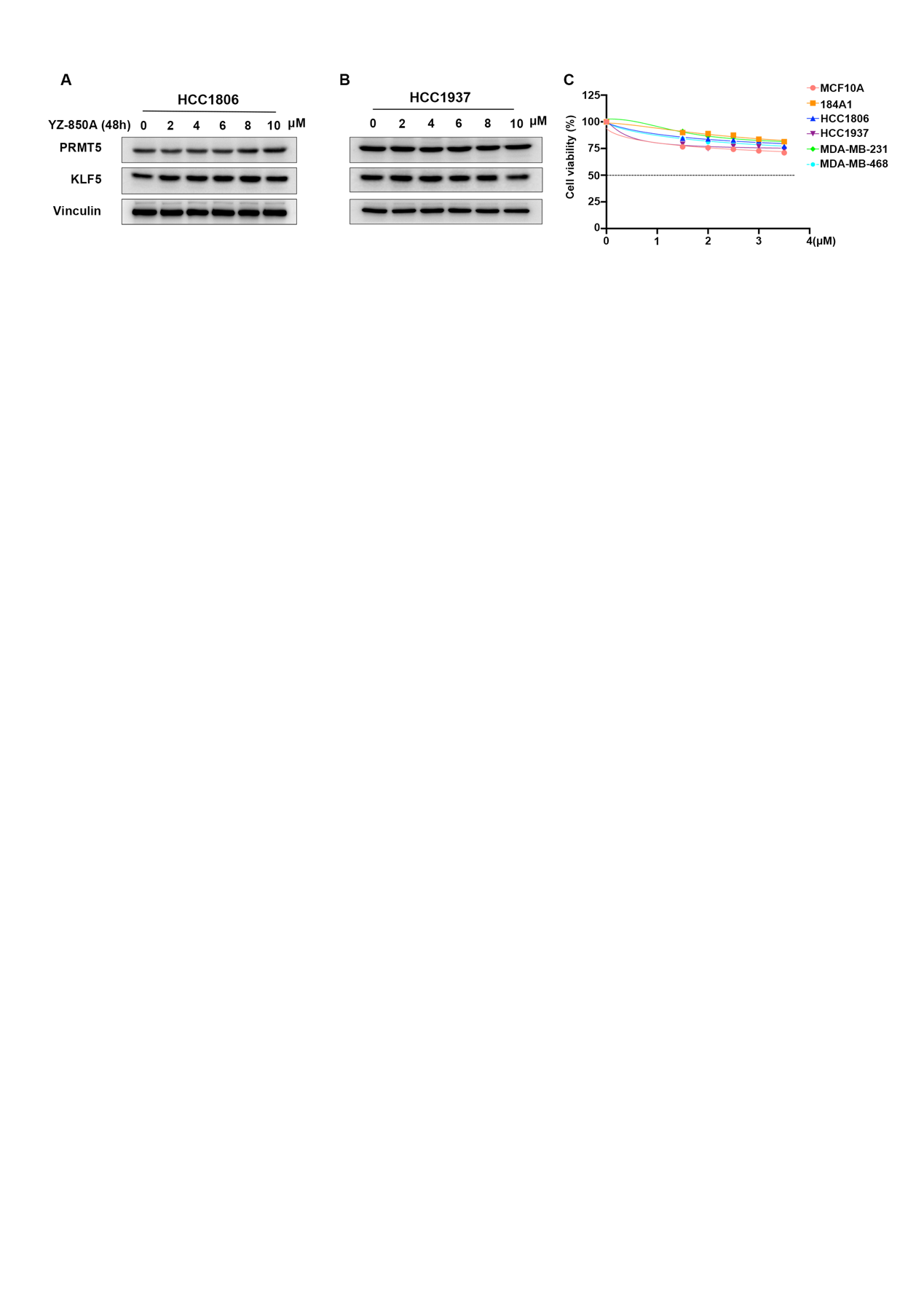
**

(A) YZ-850A could not reduce PRMT5 and KLF5 protein levels in HCC1806 cell line, as determined by WB.

(B) YZ-850A could not reduce PRMT5 and KLF5 protein levels in HCC1937 cell line, as determined by WB.

(C) YZ-850A reduced the viability of various TNBC cell lines and immortalized breast epithelial cell lines slightly.

**Figure S4. CRBN knockdown in TNBC cells reverses the cytotoxic effects of YZ-836P**

**
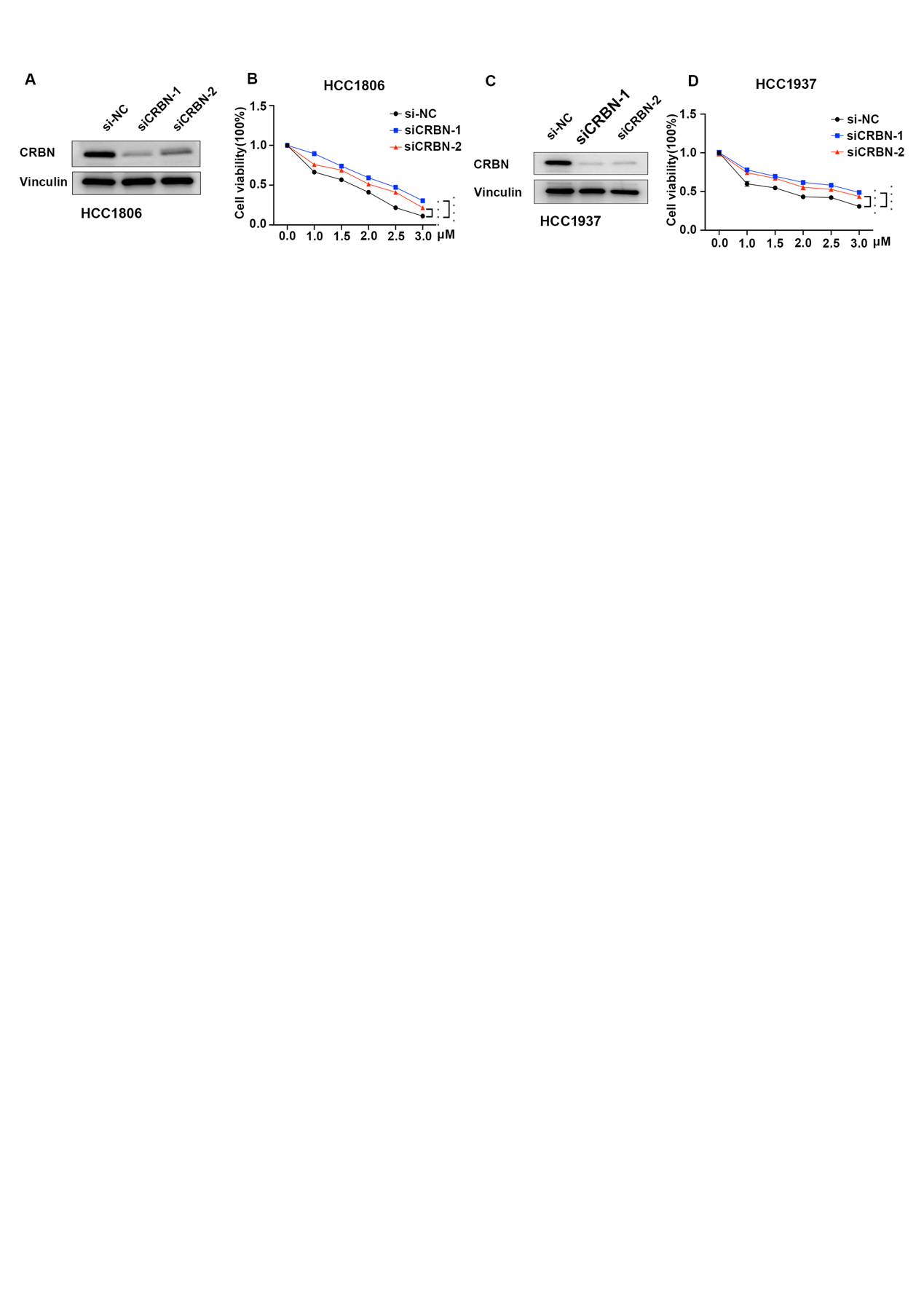
**

(A) WB showing CRBN knockdown in HCC1806 cells.

(B) CRBN knockdown in HCC1806 cells reversed the cytotoxic effects of YZ-836P (48 h).

(C) WB showing CRBN knockdown in HCC1937 cells.

(D) CRBN knockdown in HCC1937 cells reversed the cytotoxic effects of YZ-836P (48 h).

**Figure S5. PRMT5 PROTAC YZ-836P inhibits TNBC**


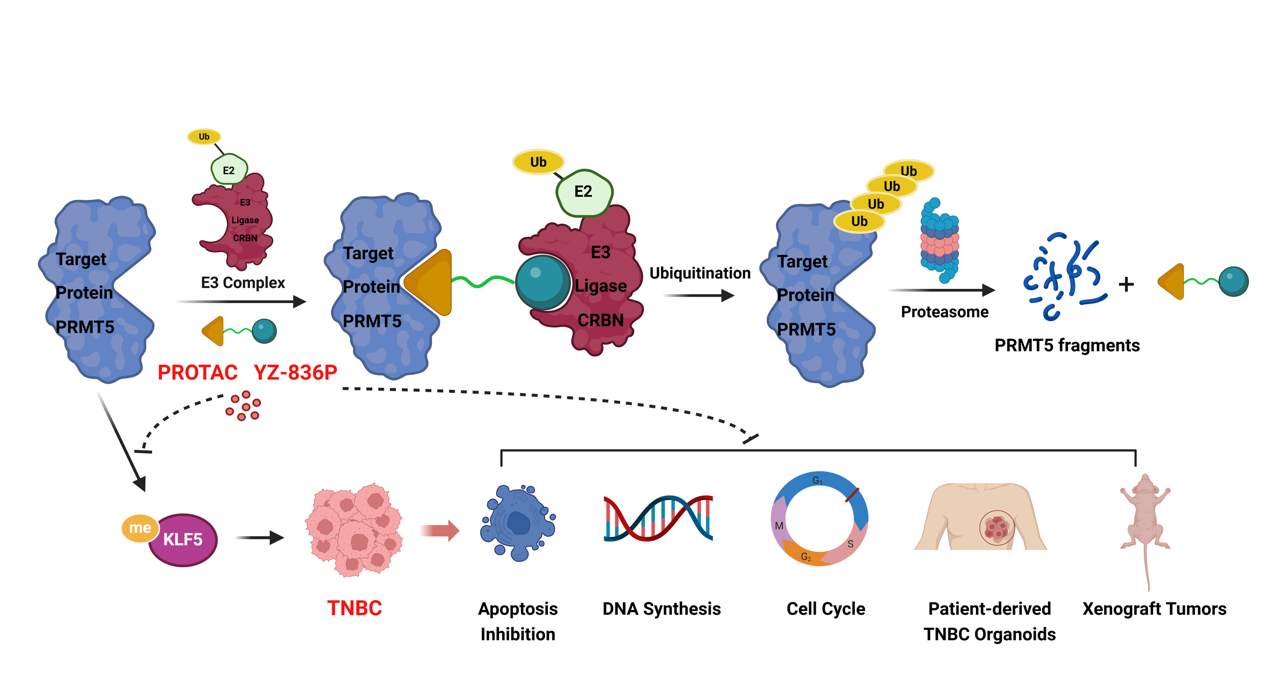


YZ-836P ingeniously combines a PRMT5-specific ligand with a ligand for the E3 ubiquitin ligase CRBN. By fostering physical proximity between PRMT5 and CRBN, YZ-836P forms a stable complex within cells, thereby facilitating the effective degradation of PRMT5 via the proteasome system. Concurrently, this PROTAC reduces the protein levels of PRMT5's key downstream target, KLF5 protein. Notably, YZ-836P inhibits the growth of TNBC cells, induces cell cycle arrest at the G1 phase, and significantly increase apoptosis. Furthermore, YZ-836P effectively impedes the growth of PDOs and markedly slows the progression of xenograft tumors *in vivo.*

**Figure S6. ^1^H NMR spectra of compound YZ-836P and YZ-850A**

**
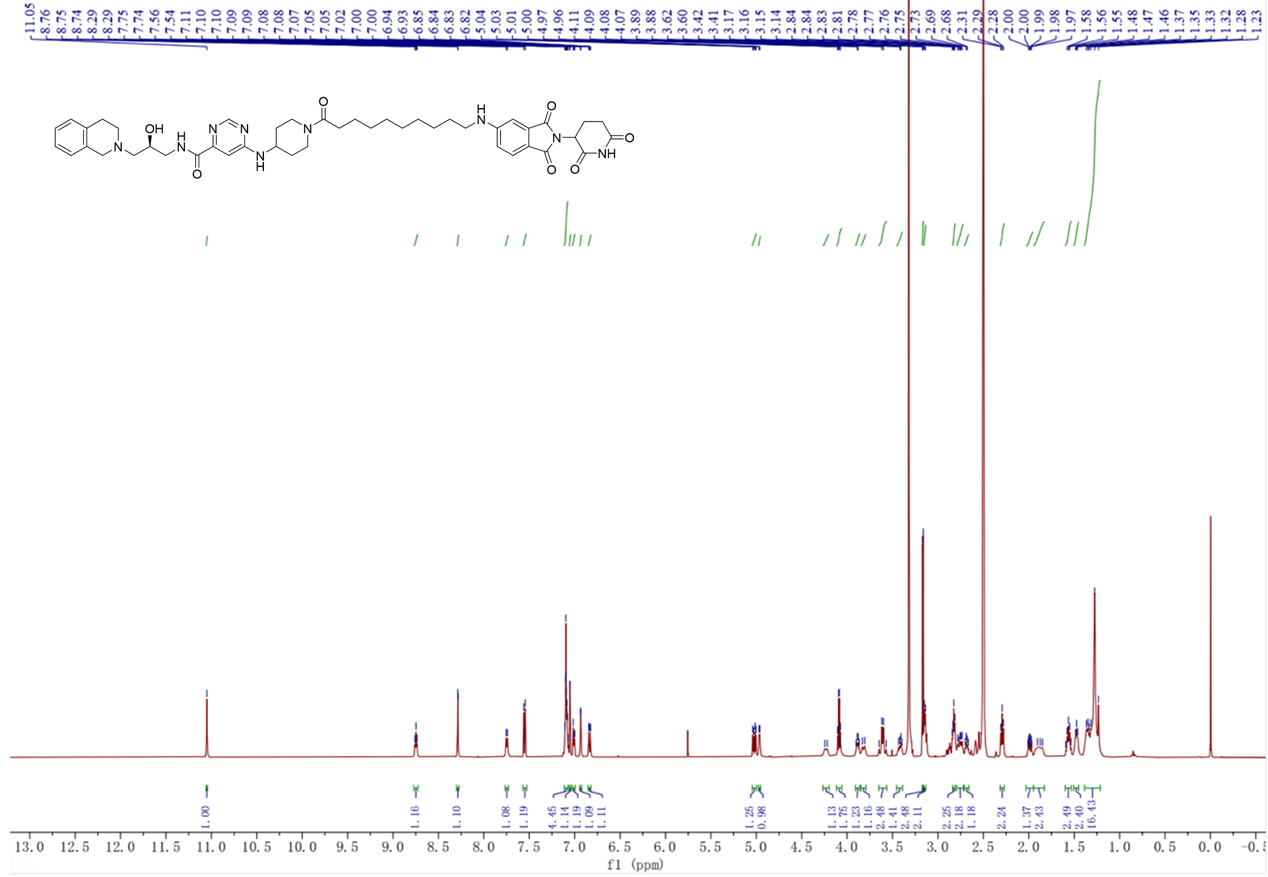
**

**
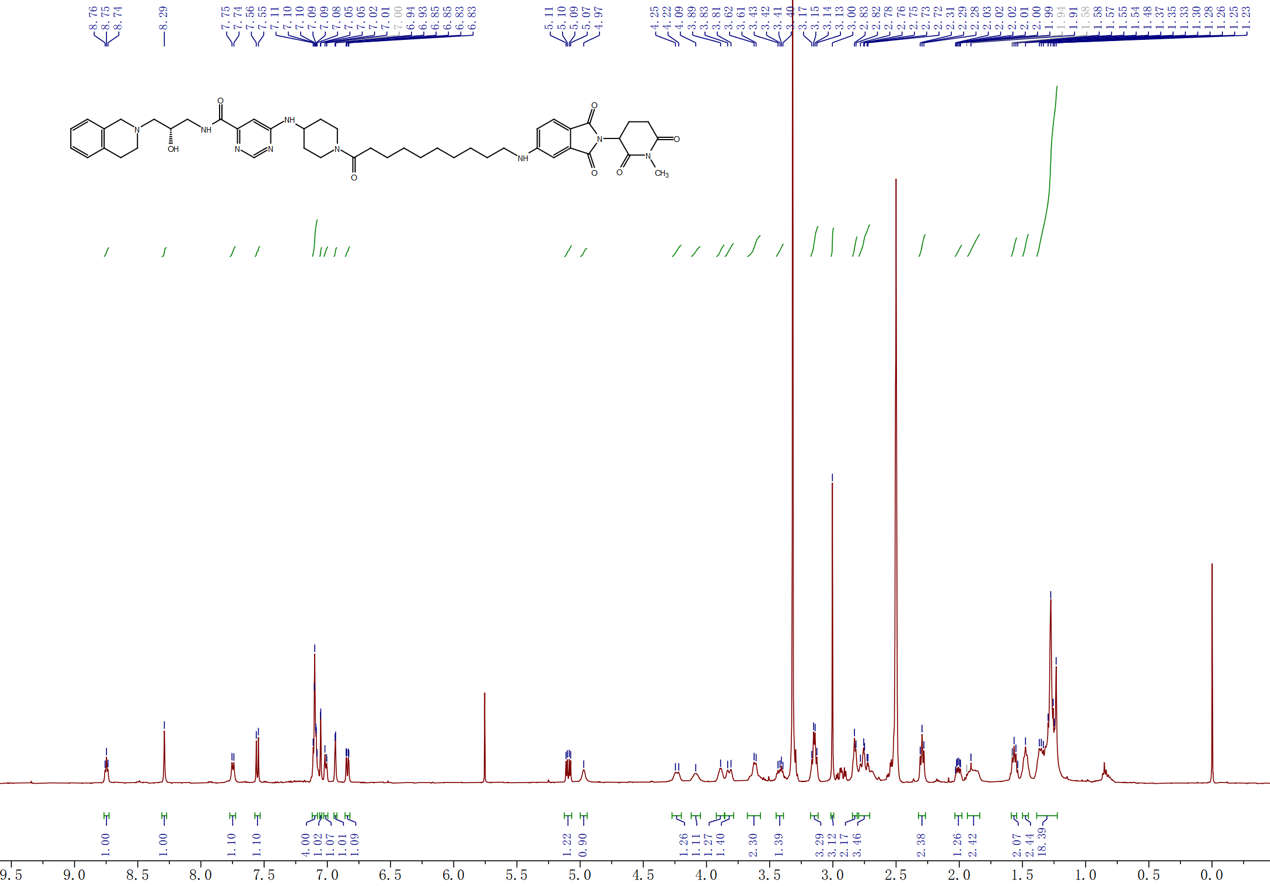
**

**Figure S7. HRMS spectra of compound YZ-836P and YZ-850A**

**
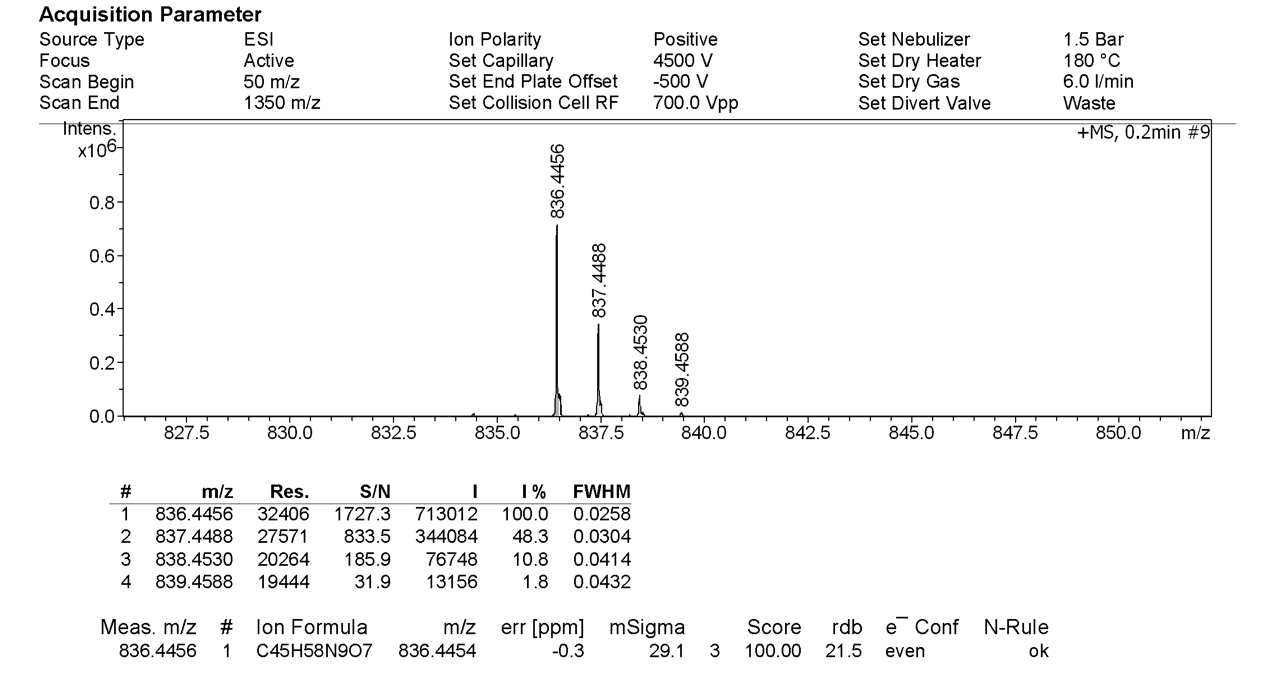
**

**
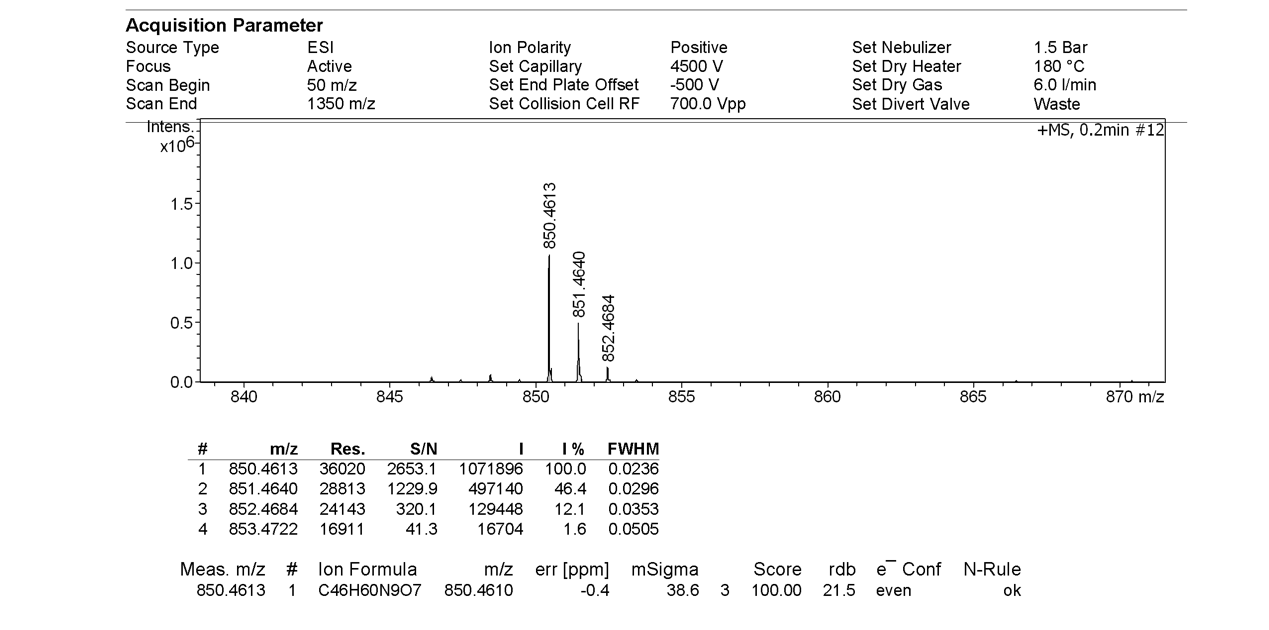
**

**Figure S8. HPLC spectra of compound YZ-836P**


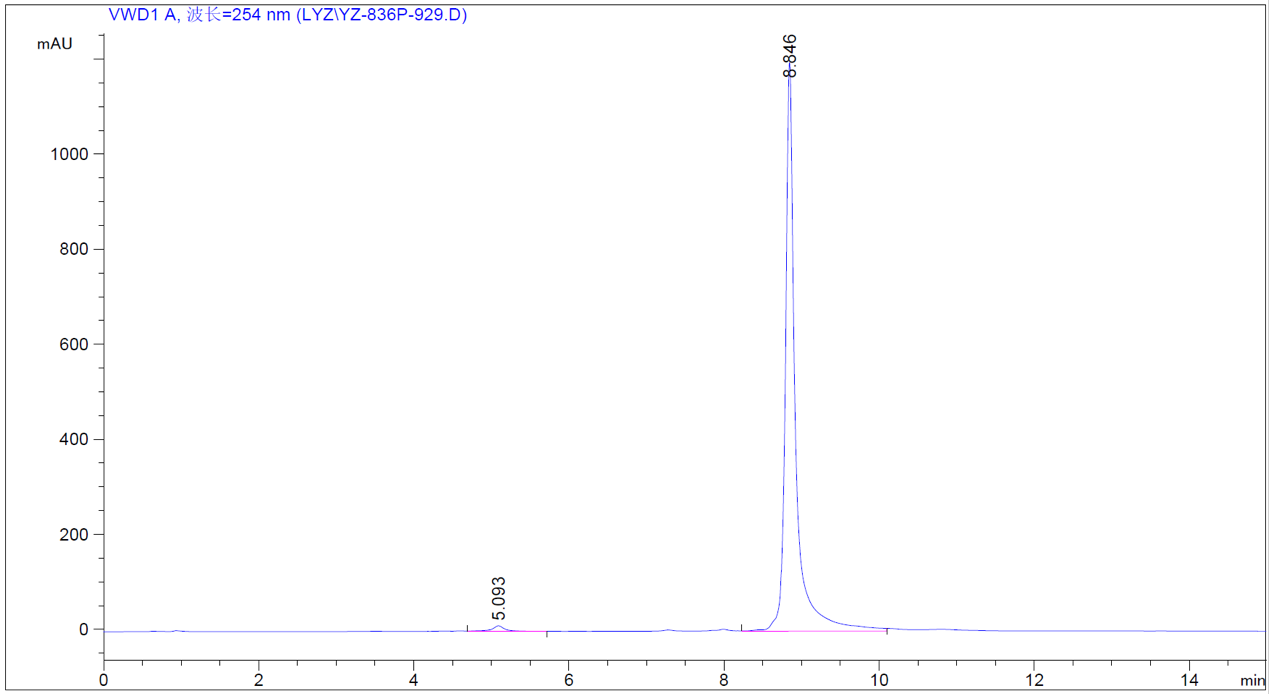


| Peak^#^ | Ret Time  (min) | Width  (min) | Area  (mAU*s) | Area% |
| --- | --- | --- | --- | --- |
| 1 | 5.093 | 0.1721 | 149.33 | 1.35 |
| 2 | 8.846 | 0.1296 | 1.09e4 | 98.65 |

Table S1. List of medium formula used in this study

| Cells | Medium |
| --- | --- |
| HCC1806 | RPMI1640, 5% FBS |
| HCC1937 |
| BT474 |
| SKBR3 |
| MDA-MB-231 | DMEM/F12,10% FBS |
| MDA-MB-468 | DMEM+10% FBS |
| MCF-7 |
| HEK-293T |
| T47D | RPMI1640+10% FBS, 0.2 Units/mL insulin |
| MCF10A | DMEM F12+5% horse serum, 100 ng/mL cholera toxin,  0.16 Units/mL insulin, 20 ng/mL epidermal growth factor, 500 ng/mL hydrocortisone |
| 184A1 |

Table S2. List of antibodies used in this study

| Antibodies | Cat No | Company |
| --- | --- | --- |
| PRMT5 | 18436-1-AP | Proteintech |
| KLF5 | #40674 | Cell Signaling Technology |
| Cleaved Caspase-3 | #9579 | Cell Signaling Technology |
| Vinculin | V9131 | Sigma-Aldrich |
| CRBN | #71810 | Cell Signaling Technology |
| CDK4 | sc-749 | Santa Cruz Biotechnology |
| CDK6 | #3136 | Cell Signaling Technology |
| Cyclin D1 | A11022 | ABclonal |
| p21 | sc-397 | Santa Cruz Biotechnology |
| p27 | #3686 | Cell Signaling Technology |
| PARP | #9542 | Cell Signaling Technology |
| Caspase3 | #9662 | Cell Signaling Technology |
| Mcl-1 | #94296 | Cell Signaling Technology |
| XIAP | #14334 | Cell Signaling Technology |

Table S3. List of primer sequences used in this study

| Primers | Primer sequences（5’-3’） | Company |
| --- | --- | --- |
| PCDH-PRMT5-F | TAGTCCAGTGTGGTGGAATTCGCGGCGATGGCGGTCGGG | Tsingke |
| PCDH-PRMT5-R | GGCCGCTTAGATATCCTCGAGCTAGAGGCCAATGGTATATGAGCG | Tsingke |
| PCDH-Flag-KLF5-F | TTCTGTATGAGACCACGCGTATGGCTACAAGGGTGCTG | Tsingke |
| PCDH-Flag-KLF5-R | CGATAAGCTTGGAGACGCGTTCAGTTCTGGTGCCTCTT | Tsingke |
| PCDH-Flag-PRMT5-F | GATGACAAGTCTAGAGAATTCGCGGCGATGGCGGTCGGG | Tsingke |
| PCDH-Flag-PRMT5-R | GTAAAGCTTCCATGGCTCGAGCTAGAGGCCAATGGTATATGAGCG | Tsingke |
| PCDH-CRBN-F | TTTTGACCTCCATAGAAGATTCTAGAATGGCCGGCGAAGGAGATCAGCAG | Tsingke |
| PCDH-CRBN-R | GATCGCAGATCCTTCGCGGCCGCTTACAAGCAAAGTATTACTTTGTCTGGACTTATTTC | Tsingke |

**Table S**4. List of siRNAs used in this study

| siRNAs | sequence | Company |
| --- | --- | --- |
| siCRBN-1# | GTAGCTGCTTGTCTTCCTA | RIBOBIO |
| siCRBN-2# | GAAGCACAGTTTGGAACAA | RIBOBIO |
